# Trends in self-reported dilated eye examinations among diabetic adults in California from 2011 to 2021

**DOI:** 10.1371/journal.pone.0336246

**Published:** 2025-12-23

**Authors:** Xiaoli Niu, Bruce Burkemper, Xuejuan Jiang

**Affiliations:** 1 Department of Population and Public Health Sciences, Keck School of Medicine, University of Southern California, Los Angeles, California, United States of America; 2 Department of Ophthalmology, Keck School of Medicine, University of Southern California, Los Angeles, California, United States of America; University of Florida College of Medicine, UNITED STATES OF AMERICA

## Abstract

Although prior studies have documented disparities in eye care utilization by age, race/ethnicity, sex, and socioeconomic status, limited research has tracked long-term trends within the diabetic population in California, particularly in the context of the COVID-19 pandemic. We aimed to estimate the 11-year secular trend in eye care utilization among adult diabetic patients in California, USA. This was a secondary analysis of public-use data from the California Health Interview Survey (CHIS), an annual population-based survey in California conducted from 2011 to 2021. CHIS samples a representative portion of the California population, with over 20,000 adults in each survey cycle. Data analyses were conducted from January 2023 to April 2024. The primary outcome was the weighted percentage of diabetic adults who reported undergoing a dilated eye exam within the past year. Survey-weighted multivariable logistic regression analyses were conducted to evaluate the association between sociodemographic risk factors and the outcome. The prevalence of recent dilated eye exams declined by 8.3% (95% confidence interval -– CI: 4.7%−11.8%; P < 0.0001) in the two years following the onset of the COVID-19 pandemic (2020−2021) compared to the two years prior (2018−2019) among adults with diabetes in California. This decline was more pronounced among adults aged ≥ 60 years (P = 0.040 for age interaction) and was limited to urban residents (P = 0.030 for interaction with geographic area). However, it did not disproportionately affect Hispanic or Black Americans (P = 0.71 for race/ethnicity interaction). In conclusion, eye care utilization among the diabetic adults in California declined significantly from 2018−2019–2020−2021, a trend largely attributable to the impact of the COVID-19 pandemic.

## Introduction

Diabetic patients have a substantially greater risk of blindness compared to those without diabetes [1]. Regular eye examinations and treatments are crucial for diabetic patients. However, eye care utilization among this population varies significantly by factors such as age, race/ethnicity, and education [2]. Additionally, health policy changes, such as the Affordable Care Act (ACA) Medicaid expansion in the US, have been shown to influence eye care utilization among low-income diabetic patients [3]. The onset of the COVID-19 pandemic and the ensuing policy changes could also significantly impact eye care utilization. In early 2020, the American Academy of Ophthalmology (AAO) recommended that ophthalmologists cease providing non-urgent care, limiting services to urgent or emergent cases [4]. Furthermore, social distancing measures and the need for personal protective equipment posed challenges for eye care providers conducting routine exams [5]. However, the precise extent of the pandemic’s impact on eye care utilization at the population level remains unclear.

This study aims to assess long-term trends in the prevalence of dilated eye exams among diabetic adults in California, specifically focusing on quantifying changes before and after the COVID-19 pandemic. Furthermore, we seek to evaluate disparities in this trend across various sociodemographic factors, including race/ethnicity.

## Materials and methods

### Study design and data collection

We analyzed the public-use data from the California Health Interview Survey (CHIS), specifically from the Adult Computer-assisted Web Interviewing Questionnaire, collected between 2011 and 2021. CHIS datasets were downloaded from https://healthpolicy.ucla.edu/our-work/public-use-files on Jan 26, 2023. CHIS is a population-based survey conducted annually since 2011, employing a multimode (online and telephone) approach to assess California’s residential, noninstitutionalized population. CHIS utilizes an address-based sampling design to ensure statewide representation. The authors do not have access to information that could identify CHIS participants. The project was reviewed and determined to be exempt under the Federal Regulations for Protection of Human Research Subjects (45 CFR 46) by the University of Southern California Institutional Review Board.

### Outcome definition

The history of dilated eye exams was assessed based on self-reported responses to the question: “*When was the last time you had an eye exam in which the pupils were dilated? This would have made your eyes sensitive to bright light for a short time*”. Participants who reported undergoing a dilated eye exam within the past month or the past year were classified as having had a dilated eye exam within one year.

### Sociodemographic variables

Race and ethnicity in CHIS were categorized using the Office of Management and Budget classification: Hispanic, Non-Hispanic (NH) white, African-American (NH), Asian (NH), American Indian/Alaskan Native (NH), and Other races. Age was categorized as <40, 40–59, and ≥60 years based on the Nation Eye Institute’s recommendation on the starting age for dilated eye examination [6]. Educational attainment was categorized as: less than high school, high school diploma, some college, college degree, some graduate school or higher. Urban versus rural geographic designation was determined by zip code using Claritas geographic classification data.

### Statistical analyses

Our analysis was limited to CHIS adult participants with diabetes. Individuals with diabetes were identified based on their responses to the question: “Has a *doctor ever told you that you have diabetes (non-gestational)?*” Responses indicating “yes” were categorized as diabetic, while responses indicating “pre-diabetes and borderline” or “no” were excluded.

CHIS employs the replicate weight method, which accounts for the complexities of the sampling design and provides a more accurate variability estimation. We pooled cross-sectional survey data from 2011 to 2021 into a single dataset. New replicate weights were generated to accommodate pooling across survey cycles, as recommended by CHIS. The chi-square test with the Rao–Scott second-order correction was used to examine differences in the prevalence of dilated eye examinations across subgroups and across before and after the COVID-19 pandemic (2020–2021 vs. 2018–2019). For comparison purpose, we also assessed differences before and after the implementation of the ACA (2014–2017 vs. 2011–2013). Interaction terms were tested by using replicate logistic multivariable regression. We applied the design-based jackknife method for variance estimation to replicate weights to obtain state-representative results.

All statistical analyses were conducted using SAS 9.4 (SAS Institute Inc., Cary, NC, USA). The significance level was set as P ≤ 0.05.

## Results

Between 2011 and 2021, 236,156 adults participated in CHIS. Of these participants, 27,089 had non-gestational diabetes. The mean age of these adults was 63.2 (± 13.0) years, 49.5% were male, and fewer than 20% were rural residents. Table 1 presents the demographic characteristics of people with diabetes in CHIS from 2018 to 2021.

**Table 1:** Distribution of demographic characteristics among diabetic participants in California Health Interview Survey (CHIS), years 2018–2021.

Characteristic, N (%)	Year 2018	Year 2019	Year 2020	Year 2021
**Total**	2668 (100%)	2697 (100%)	2367 (100%)	2668 (100%)
**Age**				
** < 60**	729 (27.3%)	707 (28.3%)	676 (28.6%)	957 (35.9%)
** ≥ 60**	1939 (72.7%)	1790 (71.7%)	1691 (71.4%)	1711 (64.1%)
**Sex**				
** Male**	1308 (49.0%)	1375 (55.1%)	1255 (53.0%)	1430 (53.6%)
** Female**	1360 (51.0%)	1122 (44.9%)	1112 (47.0%)	1238 (46.4%)
**Race/ethnicity**				
** Hispanic**	663 (24.9%)	518 (20.7%)	572 (24.2%)	784 (29.4%)
** White (NH**^**1**^)	1411 (52.9%)	1413 (56.6%)	1258 (53.2%)	1106 (41.5%)
** African American Only (NH)**	221 (8.3%)	146 (5.9%)	141 (6.0%)	200 (7.5%)
** American Indian/Alaskan Native Only (NH)**	69 (2.6%)	18 (0.7%)	14 (0.6%)	29 (1.1%)
** Asian Only (NH)**	224 (8.4%)	341 (13.7%)	316 (13.4%)	441 (16.5%)
** Other/Two or more races**	80 (3.0%)	61 (2.4%)	66 (2.8%)	108 (4.1%)
**Education**				
** Less than high school**	371 (13.9%)	169 (6.8%)	760 (3.5%)	231 (8.7%)
** High school diploma**	563 (21.1%)	422 (16.9%)	2400 (10.9%)	430 (16.1%)
** Some college**	835 (31.3%)	893 (25.4%)	6276 (28.6%)	899 (25.3%)
** College graduate**	501 (18.8%)	633 (25.4%)	7163 (32.6%)	675 (25.3%)
** **Some graduate school or higher	398 (14.9%)	380 (15.2%)	5350 (24.4%)	433 (16.2%)
Geographic area				
** **Urban	2165 (81.2%)	2031 (81.3%)	1899 (80.2%)	2276 (85.3%)
** **Rural	503 (18.8%)	466 (18.7%)	468 (19.8%)	392 (14.7)

^1^Abbreviation: NH = Non-Hispanic.

**Fig 1:**
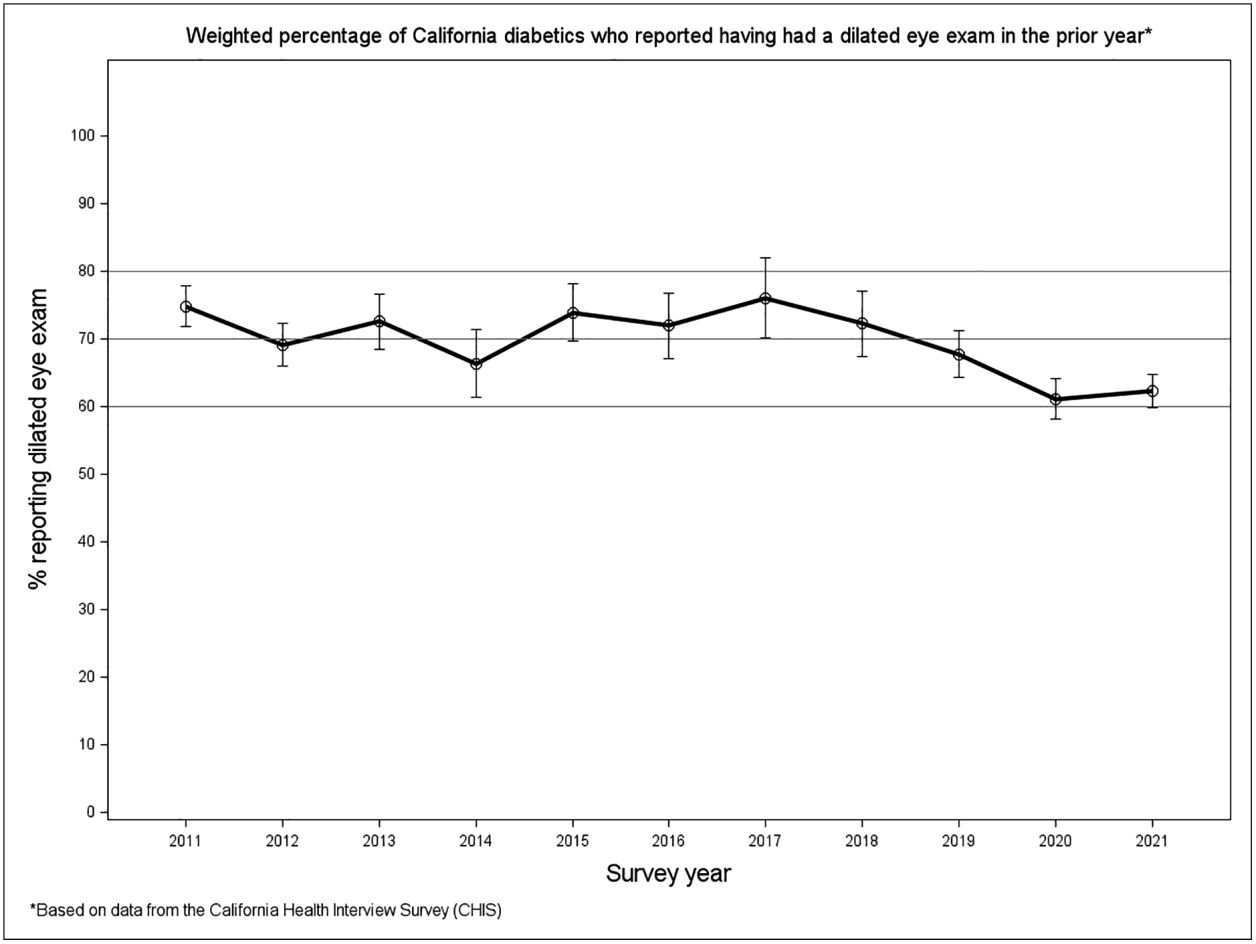
Proportions of diabetic adults having had a dilated eye exam in the prior year in California. Weighted prevalence estimates of dilated eye exams were based on data from CHIS. Error bars represent the corresponding 95% CI.

Overall, 69.2% of adults with diabetes reported having undergone a dilated eye exam in the past 12 months. Recent exams were more prevalent among individuals older than 60, non-Hispanic Whites, and those with higher education levels (Ps < 0.0001). Prevalence was similar by sex (70.2% in females vs. 69.0% in males; P = 0.28). Fig 1 shows the weighted prevalence of recent dilated eye exams across different years, ranging from 61% in 2020 to 76% in 2017. There was a significant difference across years (p < 0.0001). Among adults under age 65, the prevalence of dilated eye exam was lower immediately after the ACA (in 2014) (56.8%, P = 0.012) but returned to pre-ACA level in 2015–2017 (68.8%, P = 0.31), compared to the 3 years before the ACA (66.2% in 2011–2013).

**Fig 2:**
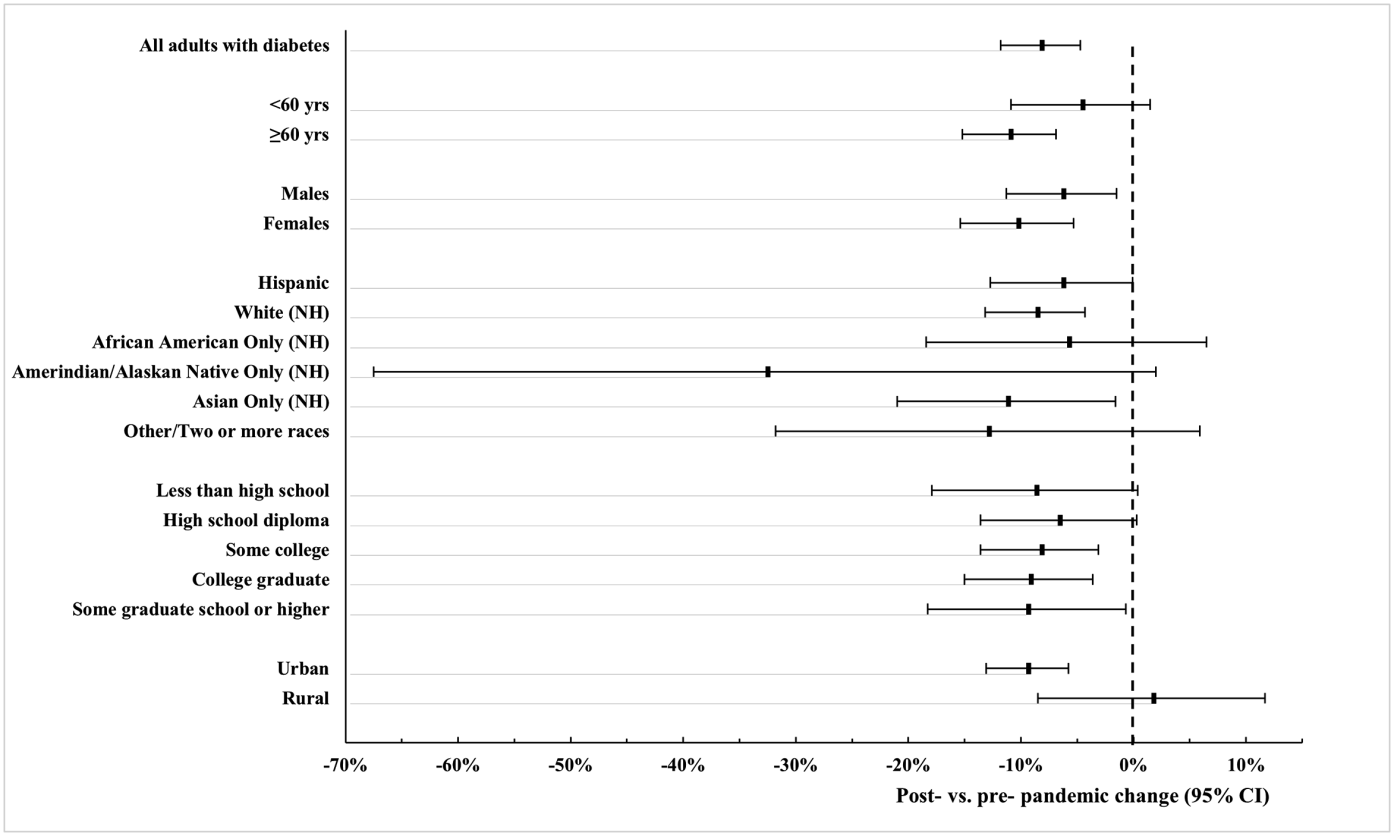
Patterns of post- vs. pre-pandemic changes in recent dilated eye exam by sociodemographic factors. Estimates were based on data from CHIS. NH = non-Hispanic. Error bars represent the corresponding 95% CI.

There was a significant drop in the prevalence of recent dilated eye exams from 70.0% (95% CI, 67.0% – 73.0%) in the 2 years before the COVID-19 pandemic (2018−2019) to 61.7% (59.8% – 63.7%) in the 2 years after (2020−2021). Notably, the 2020−2021 prevalence was not only lower than that of 2018−2019 but also below the average for the entire preceding 9-year period (2011−2019: 71.7%; 95% CI: 70.2%− 73.2%; p for difference<0.0001), making the lowest prevalence during the study period. The magnitude of change differed significantly across age groups (Fig 2, Table 2, P = 0.040 for interaction), with a greater reduction among individuals aged≥ 60 years. Racial/ethnic differences were not statistically significant (P = 0.71), although the greatest decline was observed among American/Indian/Alaskan Natives and the least among African Americans. Similarly, the pre- and post- pandemic change was not significantly different across sex and education levels (Ps for interaction >0.05). Interestingly, the decline was significant among urban residents but not among rural residents (P for interaction = 0.031).

**Table 2:** Change in Estimated proportion of diabetic residents having had a dilated eye exam in the past year after COVID-19 pandemic, overall and stratified by characteristics of interest.

*Characteristic*	Pre-COVID (2018–2019)^1^	Post-COVID (2020–2021)^1^	Post – Pre % Difference (95% CI)	P for difference^2^	P for interaction^3^
**All diabetic California residents**	70.0%	61.7%	−8.3% (−11.8% to −4.7%)	<0.0001	–
**Age**					0.040
** < 60**	60.0%	55.3%	−4.7% (−10.9% to 1.5%)	0.14	
** ≥ 60**	77.7%	66.6%	−11.1% (−15.2% to −6.9%)	<0.0001	
**Sex**					0.26
** Males**	68.8%	62.4%	−6.4% (−11.3% to −1.5%)	0.012	
** Females**	71.4%	61.0%	−10.4% (−15.4% to −5.3%)	<0.0001	
**Race/Ethnicity**					0.71
** Hispanic**	66.2%	59.7%	−6.4% (−12.7% to −0.1%)	0.049	
** White (NH)**	74.9%	66.2%	−8.7% (−13.2% to −4.3%)	0.0002	
** African American Only (NH)**	69.9%	64.0%	−5.9% (−18.4% to 6.5%)	0.36	
** American Indian/Alaskan Native Only (NH)**	81.0%	48.3%	−32.7% (−67.5% to 2.0%)	0.020	
** Asian Only (NH)**	70.2%	58.9%	−11.3% (−21.0% to −1.6%)	0.025	
** Other/Two or more races**	65.3%	52.3%	−13.0% (−31.8% to 5.9%)	0.17	
**Education**					0.95
** Less than high school**	64.7%	55.9%	−8.8% (−17.9% to 0.4%)	0.063	
** High school diploma**	68.2%	61.6%	−6.7% (−13.6% to 0.3%)	0.065	
** Some college**	72.3%	63.9%	−8.3% (−13.6% to −3.1%)	0.0030	
** College graduate**	75.0%	65.7%	−9.3% (−15.0% to −3.6%)	0.0019	
** Some graduate school or higher**	76.8%	67.3%	−9.5% (−18.3% to −0.7%)	0.043	
**Geographic area**					0.031
** Urban**	71.1%	61.6%	−9.5% (−13.1% to −5.8%)	<0.0001	
** Rural**	61.2%	62.8%	1.6% (−8.5% to 11.7%)	0.75	

^1^Proportions were estimated based on survey-weighted percentage of diabetic CHIS participants who reported having had a dilated eye exam in past year, averaged over the 2-year period.

^2^Based on t-tests with degrees of freedom equal to number of replicate weights (320). ^3^Interaction tested whether the pre- and post-COVID-19 pandemic difference was different by factors including age, sex, race/ethnicity, and level of education.

## Discussion

Using state-representative data from CHIS, we investigated trends in eye care utilization in California from 2011 to 2021. The prevalence of recent dilated eye exams among the diabetic adults fluctuated over this period. A significant 8.3% decline in dilated eye exams was observed among diabetic adults in California during the COVID-19 pandemic. This decline was more pronounced among older adults and urban residents.

Consistent with findings from other Medicaid expansion states [3], we observed a slight decline in the prevalence of dilated eye exams in California in the year following ACA implementation, followed by a return to pre-expansion levels within a few years among adults under age 65. This pattern supports the validity of CHIS data in capturing secular tends in preventive eye care.

The COVID-19 pandemic significantly impacted eye care utilization among the diabetic population. Our findings align with reported declines in primary care, ophthalmological emergencies, and dental care utilization during the COVID-19 pandemic. [7–9]. For example, data from the Behavioral Risk Factor Surveillance System (BRFSS) [9] showed a declines in annual eye exams among participants living with diabetes – from 72.2% in 2019 to 68.7% in 2021.The most significant decline was seen in those aged≥ 60 years, the group with the highest baseline utilization.

Although some studies reported uniform reductions in healthcare engagement for diabetes-related care across both metropolitan and non-metropolitan areas [9], we found that the decline in dilated eye exam was limited to urban residents. This is consistent with a BRFSS study report showing increased dilated fundus examination in 2020 among rural residents, despite an overall population-wide decline [8]. These findings emphasize the need to account for rural-urban differences when addressing diabetic eye care and related health behavior.

The pandemic disproportionately affected Hispanic and Black Americans, who experienced higher rates of testing positive than white Americans [10]. However, these groups did not experience disproportionate declines in dilated eye examination. This finding is consistent with reports that Hispanics and Black Americans were not disproportionally affected in delaying dental care during the pandemic [11].

This study has limitations. First, our study design was cross-sectional, not longitudinal. Second, data on dilated eye exams was self-reported and may be subject to recall bias. Third, we were unable to distinguish type 1 and type 2 diabetes. However, since type 1 diabetes accounts for less than 5% of all diabetes cases [12], this limitation is unlikely to affect our findings on overall trends over time. Additionally, the population of American Indian/Alaskan Natives was limited. Lastly, findings may not be generalizable beyond California.

## Conclusions

In conclusion, our analysis of self-reported dilated eye exams among diabetic adults in California from 2010 to 2021 revealed a significant decline during the COVID-19 pandemic, particularly among older adults and urban residents. These results highlight the need for targeted public health interventions and policy efforts to restore preventive eye care services in the face of future public health emergencies.

## References

[1] Singh R, Ramasamy K, Abraham C, Gupta V, Gupta A. Diabetic retinopathy: an update. Indian J Ophthalmol. 2008;56(3):178–88. doi: 10.4103/0301-4738.40355 18417817 PMC2636123

[2] Morales LS, Varma R, Paz SH, Lai MY, Mazhar K, Andersen RM, et al. Self-reported use of eye care among Latinos: the Los Angeles Latino Eye Study. Ophthalmology. 2010;117(2):207-15.e1. doi: 10.1016/j.ophtha.2009.07.015 20018380 PMC2835414

[3] Chen EM, Armstrong GW, Cox JT, Wu DM, Hoover DR, Del Priore LV, et al. Association of the affordable care act medicaid expansion with dilated eye examinations among the united states population with diabetes. Ophthalmology. 2020;127(7):920–8. doi: 10.1016/j.ophtha.2019.09.010 31735405

[4] Ahmed I, Liu TYA. The Impact of COVID-19 on diabetic retinopathy monitoring and treatment. Curr Diab Rep. 2021;21(10):40. doi: 10.1007/s11892-021-01411-6 34495377 PMC8425316

[5] Elam AR, Sidhom D, Ugoh P, Andrews CA, De Lott LB, Woodward MA, et al. Disparities in eye care utilization during the COVID-19 pandemic. Am J Ophthalmol. 2022;233:163–70. doi: 10.1016/j.ajo.2021.07.024 34324852 PMC8312151

[6] Get a Dilated Eye Exam: National Eye Institute; 2021 [updated May 19, 2021May 14, 2024]. Available from: https://www.nei.nih.gov/learn-about-eye-health/healthy-vision/get-dilated-eye-exam

[7] Ramsey DJ, Lasalle CC, Anjum S, Marx JL, Roh S. Telehealth encourages patients with diabetes in racial and ethnic minority groups to return for in-person ophthalmic care during the COVID-19 pandemic. Clin Ophthalmol. 2022;16:2157–66. doi: 10.2147/OPTH.S368972 35814918 PMC9268229

[8] Emmert R, Thompson M, Smith D, Marlar R, McPherson K, Demla S, et al. Prevalence of diabetic retinopathy and dilated fundus examinations by metropolitan status from 2017-2021: an assessment of the behavioral risk factor surveillance system. Ophthalmic Epidemiol. 2025;32(5):476–9. doi: 10.1080/09286586.2024.2434247 39693586

[9] Vashist K, Frediani JK, Weber MB, Ali MK, Narayan KMV, Patel SA. Changes in diabetes care and management practices during the COVID-19 pandemic. Res Sq. 2024;rs.3.rs-3849240. doi: 10.21203/rs.3.rs-3849240/v1 39521444 PMC13032025

[10] Magesh S, John D, Li WT, Li Y, Mattingly-App A, Jain S, et al. Disparities in COVID-19 outcomes by race, ethnicity, and socioeconomic status: a systematic-review and meta-analysis. JAMA Netw Open. 2021;4(11):e2134147. doi: 10.1001/jamanetworkopen.2021.34147 34762110 PMC8586903

[11] Kranz AM, Gahlon G, Dick AW, Stein BD. Characteristics of US adults delaying dental care due to the COVID-19 pandemic. JDR Clin Trans Res. 2021;6(1):8–14. doi: 10.1177/2380084420962778 32985322 PMC7527908

[12] Menke A, Orchard TJ, Imperatore G, Bullard KM, Mayer-Davis E, Cowie CC. The prevalence of type 1 diabetes in the United States. Epidemiology. 2013;24(5):773–4. doi: 10.1097/EDE.0b013e31829ef01a 23903880 PMC4562437

